# Supplementary material for: COVID-19 guidelines and its perceived effect on seafarers’ health and wellbeing: A qualitative study
Source: PLoS One. 2023 Apr 10;18(4):e0284155. doi: 10.1371/journal.pone.0284155 (PMC10085033; doi:10.1371/journal.pone.0284155)
Supplement: Attachment — Submitted filename: Response letter_Amit.pdf [file pone.0284155.s001.pdf]

31st October 2022

To,

The editor,

Plos One.

Subject: Regarding revision and submission of response letter (PONE-D-22-16406).

Dear editors,

We would like to re-submit our original article entitled *COVID-19 guidelines and its effect on seafarers' health and well-being* after **addressing all the comments** recommended and suggested by the reviewers. The comments and feedback from the reviewers has definitely been helpful to refine this article and we would like to take this opportunity to thank both of our reviewer for valuable insights and feedback. As stated by the reviewer, this article is very noble and important to bridge the existing gap regarding implementation of COVID-19 guidelines. Thus, we would like to request for the acceptance of this article for the publication.

Also, we author declare no conflict of interest whatsoever and request to edit in the online portal as well. If the editorial team and reviewer team has any other feedback for us, we would be happy to receive and work on them.

Please find attached point to point comments' response (in red font color) attached below.

Regards,

On behalf of co-authors

Amit Timilsina

timilsinaamit@gmaill.com

Point to point response of comments (PONE-D-22-16406)

1. Please ensure that your manuscript meets PLOS ONE's style requirements, including those for file naming. The PLOS ONE style templates can be found at

Response: The style requirement as suggested by the editors has been addressed in the manuscript.

2. Thank you for stating the following in your Competing Interests section:

“Authors declare no competing interests exist.”

Please complete your Competing Interests on the online submission form to state any Competing Interests. If you have no competing interests, please state ""The authors have declared that no competing interests exist."", as detailed online in our guide for authors at http://journals.plos.org/plosone/s/submit-now

This information should be included in your cover letter; we will change the online submission form on your behalf.

Response: The competing interest has been edited in the cover letter and as suggested by the editor, we request to change the online submission form on our behalf.

3. Your ethics statement should only appear in the Methods section of your manuscript. If your ethics statement is written in any section besides the Methods, please delete it from any other section.

Response: The ethics statement has been mentioned now only in method section. Previously, it was mentioned in the declaration section under ethics approval and consent to participant. It has been removed from this section. Only the statement regarding approval from University has been mentioned in the declaration section.

4. Please upload a copy of Figure 1, to which you refer in your text on page 7. If the figure is no longer to be included as part of the submission please remove all reference to it within the text.

Response: Figure 1 was already submitted as a separate file in the online submission form. It definitely needs to be included as part of submission.

Additional Editor Comments:

Reviewer's Responses to Questions

5. Review Comments to the Author

Please use the space provided to explain your answers to the questions above. You may also include additional comments for the author, including concerns about dual publication, research ethics, or publication ethics. (Please upload your review as an attachment if it exceeds 20,000 characters)

Reviewer #1: Literature review is inadequate

Response: This study is nobel as there are very few research conducted on effective implementation of COVID-19 guidance's and protocols. The literatures available for this study topic are very few. Literature review for this topic was revisited and some of the new researches or updates were found and added accordingly to this paper.

Methodology

• Several unclear statements. Example is on page 4"Seafarers who were on board during or after these
response mechanisms were recruited".

Response: The methodology section has been reviewed again and appropriate changes have been made which is reflected in track change and clean version.

• The verbal informed consent was obtained from all participants before the interview and the objectives of the research were explained to them. This is generally not the case, explanation should come before consent is granted.

Response: The explanation and consent process was completed prior to interview date. However, consent was verbally retaken while starting the interview as well. The language has been reworked and made clearer.

• Triangulation with only one method of data collection is misleading.

Response: Since, the topic of this study demands review of available guidances and study related to similar topic; systematic literature review was also conducted as part of triangulation. This section has been added to the methodology section acknowledging valuable suggestion by the reviewer. Thus, IDI and in-depth literature review, two different methods of triangulation suffice methodologically and is not misleading.

• The methodology is silent on the sampling techniques used. Another flaw is the general lack of justification for the choices made.
Response: The sampling technique 'Purposive sampling' and its process have now been clearly mentioned in the manuscript.

• The triangulation of data was done by interviewing three managers and reviewing the guidelines. I don't see the review of the guidelines

Response: The short section regarding review of guideline was explained in the beginning of discussion section previously. In response to the comment, we have not written a short separate section on findings of guidance review. It has also been now explained explicitly in methodology section too.

• There is no figure 1 in the document as indicated on page 7.

Response: The figure was submitted separately in the submission form.

Presentation

• Some of the point discussed were not mentioned by the participants in the study conducted. Example, participant being denied access to medical services.

Response: Not all quotes can be included within the manuscript given the word limitation for the manuscript. However, the important excerpts for denial of medical services has been now included in the manuscript.

Others
• No implication
• No proper recommendation
• Weak conclusion.
• The language is unsatisfactory. Proofreading is required.

Response: The recommendation has been updated within the discussion section, conclusion has been revised and re-written, implication of this study has been well explained at the end of discussion section and in the conclusion section. Similarly, proof-reading has been made by us and also reviewed by a professional.

Reviewer #2: First, I apologize for the delay in sending my peer review. This study is a qualitative study of the impact of the COVID-19 guidelines on the health and well-being of seafarers, and given that the COVID-19 guidelines affect people in a variety of occupations, we assert that this qualitative study is important. However, I believe that the paper would be even better if the results from this qualitative study were presented in a model. Also, would it be difficult to present the details of each of the 13 interviewees in a table, rather than presenting them as a group, e.g., "age: 29-42"?

Response:

The figure 1 illustrates the model of the study which is based on one generic research statement, two themes and six subthemes.

Having detailed information for each interviewees may lead to compromised anonymity of the participants, thus we chose to keep it as a range rather than individual socio-demographic information. For example, a seafarer of age 32 working at desk of 8 years of experience might help other know the participant. Thus, we want to anoynymize participants' information.

---

6. PLOS authors have the option to publish the peer review history of their article (what does this mean?). If published, this will include your full peer review and any attached files.

If you choose "no", your identity will remain anonymous but your review may still be made public.

Response: Yes, our identity can be revealed.
